# ¿Nuevos modelos de gestión o gestión tradicional de los hospitales? Reversión de un hospital-fundación en una eurorregión

**DOI:** 10.23938/ASSN.1100

**Published:** 2024-12-21

**Authors:** Juan Cueva Ares, Mateo Cacho Uzal, Fe Lopez-Juiz, Francisco Reyes-Santías

**Affiliations:** 1 Universidad Carlos III Facultad de Economía Departamento de Economía Madrid España; 2 Ministerio de Justicia Tribunal Superior de Justicia de Galicia A Coruña España; 3 Universidad de Vigo Facultad de Derecho Departamento de Derecho Público Vigo España; 4 Universidad de Vigo Facultad de Ciencias Empresariales y Turismo Departamento de Organización de Empresas y Marketing Vigo España; 5 Centro de Investigación Biomédica en Red en Enfermedades Cardiovasculares (CIBERCV) España; 6 Fundación Instituto de Investigación Sanitaria (FIDIS) Santiago de Compostela España

**Keywords:** Gestión Sanitaria, Hospitales Públicos, Empresas públicas hospitalarias, Eficiencia, Análisis envolvente de datos, Health Care Management, Hospitals Public, Public Hospital Enterprises, Efficiency, Data envelopment analysis

## Abstract

**Fundamento:**

Evaluar el impacto de la reversión de un hospital-fundación pública (gestión indirecta) a la gestión pública directa sobre la eficiencia de la gestión de sus recursos.

**Metodología:**

El hospital *Virxe da Xunqueira* (en la eurorregión Galicia-Norte de Portugal) se gestionó como fundación pública hasta 2010, año en que pasó a ser centro gestor del Servicio Gallego de Salud. Se comparó la eficiencia de las gestiones indirecta (2005 a 2009) y directa (2011-2015) de los recursos del hospital mediante análisis envolvente de datos. Se consideraron como *inputs* los factores trabajo (número de trabajadores) y capital (número de camas) y como *outputs* el número de consultas, urgencias, intervenciones, ingresos, estancias y pacientes en lista de espera, la duración media de la estancia y de la espera quirúrgica, y el porcentaje de ocupación; el índice sintético fueron las unidades básicas asistenciales.

**Resultados:**

El hospital *Virxe da Xunqueira* presentó mayor eficiencia como fundación pública (gestión indirecta) en el número de consultas, urgencias y pacientes hospitalizados, e indicadores relacionados con la estancia (número y duración media). Tras su reversión a centro gestor del SERGAS (gestión directa) fue más eficiente en intervenciones quirúrgicas y lista de espera (número de pacientes y tiempo medio de espera). Los factores de producción presentaron retornos decrecientes a escala en ambos tipos de gestión.

**Conclusiones:**

Ambos modelos de gestión presentaron mayor eficacia en distintos *inputs*. No se obtuvo evidencia suficiente para afirmar que la gestión por nuevos modelos de gestión (gestión indirecta) es más eficiente que la gestión directa.

## Abstract (en)

**Background:**

This study aims to assess the impact of transitioning a hospital/foundation from indirect management to direct management on the efficiency of hospital resource management.

**Methodology:**

Until 2010, the Virxe da Xunqueira hospital/foundation, located in the Galicia-North Portugal Euroregion, operated under indirect management. In 2010, it transitioned to direct management as a health centre within the Galician Health Service (Spain). Public management of hospital resources was compared using data development analysis for two periods: indirect management (2005-2009) and direct management (2011-2015). Inputs included labour (number of workers) and capital (number of beds), while outputs were measured by the number of consultations, emergencies, interventions, admissions (inpatients), hospital stays, patients on waiting the list, average length of stay, waiting times, and hospital occupancy rate. The synthetic index used was the basic care units.

**Results:**

*Virxe da Xunqueira* demonstrated greater efficiency as a hospital/foundation in terms of the number of consultations, emergencies, admissions, inpatients, and indicators related to patient stay (including number and average length of stay). After transitioning to direct management, the hospital showed improved efficiency in average waiting times, the number of patients on the waiting list, and the number of surgical interventions. The production factors exhibited decreasing returns to scale in both types of governance.

**Conclusions:**

Both type of management show greater efficiency in certain inputs. However, there is insufficient evidence to conclude that the previous management model (indirect management) is more efficient than direct management.

## INTRODUCCIÓN

La Ley 15/1997, de 25 de abril^1^, ofrece la posibilidad de contar con diferentes *modelos de provisión de servicios sanitarios*. De este modo, permite la prestación de servicios públicos gestionados por la Administración a la vez que incorpora nuevos modelos: la Administración convencional, los Entes públicos atípicos, el Consorcio, la Fundación privada pero creada por una entidad pública y la Fundación Pública Sanitaria. Las opciones se amplían cuando se permiten formas de gestión indirecta (sociedad laboral, cooperativa, sociedad mercantil y fundación privada); formas de gestión mixta (la gestión interesada y las sociedades de economía mixta) y formas de vinculación del sector privado con el sector público (convenio, concierto, concesión y arrendamiento).

La gestión de los centros, servicios o entidades puede ser directa o indirecta (Artículo Único, párrafo segundo del número primero^1^). La gestión de los centros de asistencia sanitaria es directa cuando se manejan con fondos propios cuya titularidad la tiene la Administración Pública. La gestión es indirecta cuando se manejan con medios ajenos, a través de la creación de entidades con titularidad pública admitidas en derecho, a través de convenios, acuerdos o contratos con personas o entidades públicas o privadas^1^. La gestión a través de entes interpuestos es directa (exposición de motivos^1^).

La prestación del servicio público por parte de centros, servicios o entidades de naturaleza o titularidad pública siempre es gestión directa, excepto cuando su titularidad sea de una Administración distinta de la que contrata^2^^,^^3^. No todos los convenios son gestión indirecta: son de gestión directa los contratos-programas, los convenios de gestión (mercado interno) y los programas de gestión convenida entre los centros sanitarios públicos y las autoridades sanitarias de las que dependen^4^.

Las fundaciones públicas tienen su propia personalidad jurídica, se establecen sin fines de lucro y brindan un servicio de salud, ya sea asistencia social o capacitación del personal para mejorar esta asistencia, buscando el beneficio de todos aquellos que tienen derecho a asistencia gratuita en el Sistema Nacional de Salud^5^.

En la Comunidad Autónoma de Galicia es donde se han creado más fundaciones vinculadas al sistema público de asistencia sanitaria; de hecho, esta figura jurídica se ha aplicado tanto a los hospitales comarcales de nueva creación como a la gestión de centros monográficos como el Centro Gallego de Transfusiones, el servicio autonómico de urgencias, el centro de formación del personal al servicio del SERGAS o un ente creado para promover la cooperación con Iberoamérica en el sector de la salud. Así, se crearon las fundaciones públicas *Hospital do Barbanza*^6^, *Hospital Virxe da Xunqueira*^6^, *Urxencias Sanitarias de Galicia-061*^7^, *Hospital Comarcal do Salnés*^8^, Hospital Verín^9^ y Centro de Transfusión de Galicia^10^.

Las fundaciones públicas pueden extinguirse por las mismas razones que el resto de fundaciones privadas^11^. Las causas concretas de extinción aparecen recogidas en el artículo 31 de la Ley 50/2002, que incluye la concurrencia de cualquier otra causa recogida en el acto de constitución de la fundación o contenida en sus estatutos^12^. Esto significa que no pueden ser extinguidas por la única voluntad del órgano de gobierno, abstrayéndose a la manifestación de la voluntad del fundador y del resto de los miembros del patronato. Una fundación puede extinguirse cuando expire el plazo por el que fue constituida; cuando se hubiese realizado íntegramente el fin fundacional; cuando sea imposible la realización del fin fundacional; cuando así resulte de la fusión con otra fundación o fundaciones, y cuando concurra cualquier otra causa prevista en el acto constitutivo o en los Estatutos o en las leyes.

El debate político ha puesto en cuestión la posibilidad de que las Administraciones Públicas recuperen la gestión de servicios públicos que estaban siendo objeto de gestión por entes instrumentales^13^. Hay que recordar que las evaluaciones del *Consello de Contas* con respecto a las fundaciones sanitarias eran muy críticas con el funcionamiento del modelo^14^^,^^15^.

Nada impide que los estatutos de las fundaciones conformadas por entidades públicas estipulen que, en el supuesto de extinción, el destino de los bienes de la fundación sea la Administración entendida como sujeto fundacional^12^. De hecho, el artículo 4 del Decreto 183/2008^16^ indica que: 1) los bienes, títulos o derechos pertenecientes a cada fundación extinta, pasarán a integrarse en el patrimonio de la Xunta de Galicia, y quedarán adscritos al Servicio Gallego de Salud, y 2) la extinción de las fundaciones no supondrá la cesación de la actividad asistencial realizada en los centros sanitarios en los que estaban establecidas, que continuarán formando parte de la red del Sistema Público de Salud de Galicia, pasando a integrarse como centros de gestión del Servicio Gallego de Salud. Dicho Decreto 183/2008 pretendía conducir el proceso de extinción de las fundaciones de hospitales públicos para conseguir una mayor homogeneidad del Sistema Público de Salud de Galicia a través de la mejora de las condiciones de los profesionales y de la calidad de la atención sanitaria^16^.

En este marco, el Gobierno de la Xunta de Galicia, en su consejo del treinta y uno de julio de dos mil ocho, autorizó la extinción de las siguientes fundaciones sanitarias públicas: hospitales de Barbanza, *Virxe da Xunqueira*, Verín y Salnés.

El hospital *Virxe da Xunqueira* (Cee, Pontevedra, España) es un hospital que atiende a un área con una población de 48.100 habitantes, con una densidad de población de 116 habitantes por kilómetro cuadrado; cuenta con 76 camas de hospitalización y tres quirófanos y su *case-mix* es 1,5581. Durante sus años como fundación (hasta 2010), utilizó la aplicación del pago por Grupos Relacionados de Diagnóstico (DRG) que incorpora el *case-mix* (complejidad) de los pacientes atendidos con hospitalización; la actividad ambulatoria se financió como pago por acto.

Tras el proceso de extinción, las administraciones sanitarias públicas pueden establecer mecanismos para la integración del personal de las fundaciones de salud y de las empresas públicas, directa y voluntaria, en la situación de personal estatutario con la categoría y el título académico equivalente de quien presta servicios en estos centros, instituciones o servicios con la situación de funcionario de carrera o bajo la figura del contrato laboral fijo. Las personas trabajadoras pasarán a participar de los derechos y deberes del personal estatutario en condiciones de homogeneidad para que el derecho de opción de integración sea real y efectivo en el ámbito estatutario, tendiendo a evitar las discriminaciones derivadas de la existencia de regímenes jurídicos distintos, eludiendo la concurrencia de privilegios o perjuicios por la pertenencia a uno u otro régimen jurídico^17^. En Galicia, los criterios de los procedimientos de incorporación del personal de las fundaciones públicas al Servicio Gallego de Salud (SERGAS) los establece el Decreto 91/2007^18^.

El Análisis Envolvente de Datos (DEA) es el método de evaluación de la eficiencia en hospitales más utilizado en la investigación sanitaria^19^. Combina la construcción de una frontera de eficiencia no paramétrica con los numerosos insumos y productos asociados a la producción hospitalaria; no requiere información sobre preferencias, precios, prioridades o tecnologías, y proporciona puntos de referencia y la identificación de las mejores prácticas^20^.

Diferentes estudios lo han empleado en la subregión Galicia de la región europea Galicia-Norte de Portugal, pero ninguno de los hospitales del SERGAS analizado era Fundación pública^21^^,^^22^^,^^23^ En España se ha aplicado la metodología DEA para comparar los niveles de eficiencia de hospitales que adoptaron nuevos modelos de gestión (fundaciones públicas, empresas públicas, concesiones, consorcios)^21^^,^^24^ pero no para compararla antes y después de su reversión, y en un caso porque esta todavía no se había producido^25^. Tampoco se compara la eficiencia pre y post reversión en dos estudios de casos: la atención primaria en Suecia y los hospitales en España^26^^,^^27^ Daniel Geffner sí comparó recursos y actividad antes y después de la reversión de los modelos hospitalarios de concesión sanitaria en la Comunitat Valenciana, pero sin presentar un modelo que evaluara dicho conjunto de datos^25^.

El modelo DEA se aplicará con orientación a los *outputs*, es decir, dado el nivel de *inputs*, buscará el mayor incremento proporcional de *outputs* permaneciendo dentro de la frontera de posibilidades de producción. El hospital será considerado eficiente si, y solo si, no es posible incrementar las cantidades de *output* manteniendo fijas las cantidades de *input* utilizadas, y asumiendo rendimientos constantes a escala (el incremento porcentual del *output* es igual al incremento porcentual de los recursos productivos)^19^. Las unidades eficientes serán aquellas que obtengan un coeficiente de eficiencia igual a 1 y serán ineficientes aquellas que obtengan coeficientes entre 0 y 1 (según las condiciones de Pareto-Koopmans)^28^.

Hay una ausencia de estudios que analicen la eficiencia de las organizaciones hospitalarias que desde una nueva forma de gestión hayan transitado a hospitales centros gestores de los servicios regionales de salud.

Por ello, este estudio pretende analizar los cambios en la eficiencia técnica pura (entendida como la utilización óptima de factores productivos) del servicio sanitario en el caso de la reversión del hospital *Virxe da Xunqueira*, gestionado desde 1997 como fundación pública autonómica (gestión indirecta) y que en 2010 se integró en el sistema sanitario autonómico SERGAS (gestión directa), en la eurorregión Galicia-Norte de Portugal.

## MATERIAL Y MÉTODOS

### Material

Puesto que el enfoque del análisis envolvente de datos para la medición de la eficiencia se basa en el aprovechamiento de *inputs* físicos para proporcionar una serie de *outputs*, los *inputs* empleados fueron: el número de camas (CAM, que es la variable proxy del factor capital más utilizada en la literatura)^20^^,^^29^^,^^30^^,^^31^ y el número de trabajadores a tiempo completo del hospital (TBJ), interpretado como factor *trabajo*^32^^,^^33^). Los *outputs* con los que se ha evaluado la eficiencia están relacionados con el resultado *producción*, y son: el número de consultas (CONS), el número de urgencias atendidas (URG); el número de intervenciones quirúrgicas (INTERV), el número de pacientes ingresados (INGRESOS), el número de hospitalarias (ESTANC), la duración en días de la estancia media (EST:MED), el porcentaje de ocupación del hospital (%OCUP), el número de pacientes en lista de espera (PAC), y el tiempo en días de espera quirúrgica medio (TEM)^24^.

Para la obtención de los datos relevantes, se recurrió a las publicaciones del *Consello de Contas* sobre fiscalización selectiva y resolución de listas de espera. En ellas, se informa sobre la situación de la lista de espera, e incluye información cuantitativa de gran interés. Igualmente, se utilizaron las memorias proporcionadas por los hospitales del área de A Coruña, recopiladas por la Gerencia de Gestión Integrada de A Coruña (XXIAC).

### Métodos

Con la intención de operar con un índice sintético del *output*, los datos fueron agregados y estandarizados con el uso de UBAS (Unidad Básica Asistencial) antes de la estimación de la eficiencia, es decir, se adaptó una escala que permitiera utilizar datos de distinta naturaleza. Las estancias fueron ponderadas por el valor 1, las urgencias por 0,5, al igual que las primeras consultas y, por último, las consultas sucesivas por 0,25^34^.

Con el fin de simplificar y facilitar la interpretación de los resultados, se calculó un promedio de eficiencia para los años 2005 a 2009, en el caso de la gestión como fundación, y otro promedio para los años 2011 a 2015, que corresponden al período posterior a la reversión del hospital al SERGAS como centro gestor. Se estableció un intervalo de cinco años como centro gestor del SERGAS para garantizar una comparación más homogénea tras completar el proceso de reversión.

Respecto a la metodología empleada en el propio DEA, se utilizó un código en el software R Commander (Tabla 1) que genera un vector de eficiencias (eff) que se encuentra incorporado a las tablas presentadas en la sección de Resultados. De este modo, los vectores de eficiencia se obtienen fácilmente importando los datos de los *inputs*, que permanecen constantes durante el análisis, y de los *outputs* requeridos en cada caso^35^. Los modelos propuestos en este trabajo están orientados al *output*.

**Tabla 1:** Fórmulas empleadas para realizar el Análisis Envolvente de Datos (DEA)

Código aplicado en R para la obtención del vector de eficiencias
library(Benchmarking)
library(psych)
x<- with(DATA, cbind(I1,I2))
y<- matrix(DATA$O1)
ccr<- dea(x,y, RTS=”crs”, ORIENTATION = “in”)
eff(ccr)
*Transformación previa*
$$\mu =t\mu_{r}\;,\;\mu_{r}=t\mu_{r}\;,\;\gamma_{i}=t\gamma_{i}\gamma_{i}=t\gamma_{i}\to t={\left(\sum_{i}\gamma_{i}\chi_{io}\right)}^{-1}t={\left(\sum_{i}\gamma_{i}\chi_{io}\right)}^{-1}$$
*Modelo*
$$e_{0}e_{0}=\max\sum_{r}\mu_{r}\gamma_{r0}\sum_{r}\mu_{r}\gamma_{r0}$$
$$\;s.t.\;\sum_{i}\gamma_{i}\chi_{io}=1\sum_{i}\gamma_{i}\chi_{io}=1$$
$$\sum_{r}\mu_{r}\gamma_{rj}-\sum_{i}\gamma_{i}\chi_{io}\sum_{r}\mu_{r}\gamma_{rj}-\;\sum_{i}\gamma_{i}\chi_{io}\leq 0,\;\forall j\forall j$$
$$\mu_{r},\;\gamma_{i}\;\geq \;\varepsilon ,\;\forall r,\;{i\mu }_{r},\;\gamma_{i}\;\geq \;\varepsilon ,\;\forall r,\;i$$
*Por dualidad*
$$Min\;\theta_{0}-\varepsilon \left(\sum_{r}S_{r}^{+}+\sum_{i}S_{i}^{-}\right)Min\;{\;\theta }_{0}-\;\varepsilon \left(\sum_{r}S_{r}^{+}+\sum_{i}S_{i}^{-}\right)$$
$$\sum_{j}{ℶ}_{j}\;\chi_{ij}+S_{i}^{-}=\theta_{0}\chi_{io}\;,\;i=1,\;\ldots \;,\;m\sum_{j}{ℶ}_{j}\;\chi_{ij}+S_{i}^{-}=\;\theta_{0}\chi_{io}\;,\;i=1,\;\ldots \;,\;m$$
$$\sum_{j}{ℶ}_{j}\;\gamma_{rj}+S_{r}^{+}=\;\gamma_{r0}\;,\;r=1,\;\ldots \;,\;s\sum_{j}{ℶ}_{j}\;\gamma_{rj}+S_{i}^{+}=\;\gamma_{r0}\;,\;r=1,\;\ldots \;,\;s$$
$${ℶ}_{j},\;S_{i}^{-},S_{r}^{+}\geq 0,\;\forall i,\;j,\;r{ℶ}_{j},S_{i}^{+},\;S_{r}^{+}\geq 0,\;\forall i,\;j,\;r$$

Las líneas de código se basan en un sólido fundamento teórico^19^^,^^36^ basado en la teoría de la programación fraccional. El objetivo principal es la resolución de un programa de optimización lineal, fundamentado en la maximización del valor total de los *outputs*, sujeto a ciertas restricciones. Estas restricciones incluyen que el valor total de los *inputs* tiene que ser igual a 1 (las restricciones de factibilidad), la diferencia entre el valor total de los *outputs* y el valor total de los *inputs* debe ser menor o igual a 0, y los vectores de precios han de ser mayores o iguales al valor en el arquimediano, denotado cómo “ε” (Tabla 1).

Se evalúa un modelo DEA para cada *output* con los *inputs* de número de trabajadores del hospital (TBJ) y de número de camas (CAM).

$$IMo\;\left(\chi^{t+1},\gamma^{t+1},\;\chi^{t},\;\gamma^{t}\right)={\left[\left(\frac{D_{0}^{t}\left(\chi^{t+1},\gamma^{t+1}\right)}{D_{0}^{t}\left(\chi^{t},\;\gamma^{t}\right)}\right)\left(\frac{D_{0}^{t+1}\left(\chi^{t+1},\gamma^{t+1}\right)}{D_{0}^{t+1}\left(\chi^{t},\;\gamma^{t}\right)}\right)\right]}^{1/2}$$

Una vez calculados previamente los parámetros de eficiencia del DEA, se emplearon para calcular el índice de Malmquist (IM) a fin de medir la eficiencia dinámica (si la eficiencia relativa del hospital experimentó un progreso o decrecimiento del primer periodo t al segundo periodo t+1). El IM cuantifica los cambios en la eficiencia técnica, valorando si entre dos periodos los hospitales se alejan o se acercan respecto a su correspondiente frontera de eficiencia^19^. El IM se basa en una técnica no paramétrica (no requiere especificar una forma funcional ni estimar sus parámetros) y permite definir la frontera tecnológica o las mejores prácticas a partir de las consideradas en la muestra, así como comparar las observaciones de cada hospital con la frontera tecnológica. El IM evalúa el efecto de eficiencia y el *cacht up* o efecto de recuperación^37^ (efecto marginal que experimenta un hospital para determinar el efecto de eficiencia) y se calcula para determinar si hay un avance o retroceso en la eficiencia relativa (D_0_) del hospital del periodo t al periodo t+1, calculando la relación entre el efecto de eficiencia y el efecto marginal del periodo t (siendo *x* los *inputs* e *y* los *outputs*).

### Estimación de la Función de Producción

Para completar el estudio, se realizó la estimación de una función de producción, con el modelo Cobb-Douglas, con el objetivo de analizar la influencia de los factores trabajo y capital en la *producción* de un hospital, entendiendo esta como el número de consultas primarias y sucesivas, así como el número de estancias y urgencias atendidas agregados como UBAS. Utilizando estos *outputs* y el número de camas y trabajadores como *inputs*, respectivamente, de los factores de capital y trabajo, podemos evaluar dichas funciones de producción del hospital, sea fundación pública o centro gestor del SERGAS.

El modelo Cobb-Douglas que ha sido ajustado en este estudio es:

$$Ln\;\left(UBAS\right)=Ln\;\left(\alpha \right)+\beta_{1}\;Ln\;\left(TBJ\right)+\;\beta_{2}\;Ln\;\left(CAM\right)+\varepsilon$$

siendo UBAS=Unidad Básica Asistencial, TBJ: trabajadores del hospital a tiempo completo como factor trabajo de la función de producción; CAM: número de camas del hospital como proxy del factor capital de la función de producción.

## RESULTADOS

Siguiendo el procedimiento detallado en la sección anterior, se llevó a cabo un análisis envolvente de datos para el hospital *Virxe da Xunqueira* de los años 2005, 2006 y 2009 (gestión indirecta como fundación pública y 2011 y 2015 (gestión directa como centro gestor del SERGAS). Se aprecia una diferencia clara entre ambos tipos de gestión en relación con ciertos *outputs* (Tabla 2).

**Tabla 2:** Análisis envolvente de datos para el hospital *Virxe da Xunqueira* en ambos periodos de gestión

	Fundación pública			Centro gestor SERGAS	
	(gestión indirecta)			(gestión directa)	
	2005	2006	2009	2011	2015
*Inputs*					
CAM	6	6	5	5	4
TBJ	274	301	326	328	375
*Outputs*					
CONS	71.419	62.975	61.494	62.577	63.578
URG	16.380	17.498	17.458	16.413	16.727
INTERV	2.675	2.849	3.903	3.452	3.524
INGRESOS	3.013	2.972	3.903	2.719	3.137
ESTANC	20.154	20.504	23.385	17.845	17.749
EST.MED.	6,72	6,85	7,5	6,57	5,7
%OCUP	77%	76%	81%	64%	66%
PAC	445	418	455	287	369
TEM	63	56	40,5	40,4	39,9
*DEA*					
CONS	1	0,8818	0,8725	0,8879	0,9143
URG	1	1	1	0,9401	0,9711
INTERV	0,8154	0,7906	1	0,8844	0,9151
INGRESOS	0,9185	0,8247	1	0,6966	0,8146
ESTANC	1	0,9383	1	0,7631	0,7692
EST.MED	1	0,9103	0,8517	0,8465	0,7504
%OCUP	1	0,9568	1	0,7847	0,8222
PAC	0,772	0,7482	0,6346	1	0,7883
TEM	0,7649	0,7833	1	1	1

El índice 1 significa que el hospital era eficiente en ese año para el *output* seleccionado. CAM: número de camas; TBJ: número de trabajadores a tiempo completo; CONS: número de consultas; URG: número de urgencias; INTERV: número de intervenciones quirúrgicas; INGRESOS: número de pacientes ingresados; ESTANC: número de estancias hospitalarias; EST.MED: estancia media; %OCUP: porcentaje de ocupación hospitalaria; PAC número de pacientes en lista de espera; TEM: tiempo de espera quirúrgica medio.

Con el fin de simplificar y facilitar la interpretación de los resultados, se calculó un promedio de la eficiencia para ambos periodos de gestión: privada que permite apreciar la disparidad entre ambas modalidades de gestión en el hospital *Virxe da Xunqueira* (Tabla 3). La gestión indirecta como fundación pública fue más eficiente en el número de consultas, urgencias y pacientes ingresados (CONS, URG, INGRESOS) y en los indicadores relacionados con la estancia (número y duración media: ESTANC y EST.MED) que cuando el hospital fue centro gestor del SERGAS.

**Tabla 3:** Resultados del análisis envolvente de datos para el hospital *Virxe da Xunqueira* realizado con promedios de eficiencia para ambos períodos de gestión

	Fundación pública	Centro gestor SERGAS
	(gestión indirecta)	(gestión directa)
	(2005-2009)	(2011-2015)
CONS	0,9181	0,9011
URG	1	0,9556
INTERV	0,8687	0,8998
INGRESOS	0,9144	0,7556
ESTANC	0,9794	0,7662
EST. MED	0,9207	0,7985
%OCUP	0,9856	0,8034
PAC	0,7183	0,8941
TEM	0,8494	1

El índice 1 significa que el hospital era eficiente en ese año para el *output* seleccionado. CONS: número de consultas; URG: número de urgencias; INTERV: número de intervenciones quirúrgicas; INGRESOS: número de pacientes ingresados; ESTANC: número de estancias hospitalarias; EST.MED: estancia media; %OCUP: porcentaje de ocupación hospitalaria; PAC número de pacientes en lista de espera; TEM: tiempo de espera quirúrgica medio.

El Anexo 1 recoge los datos anuales del hospital *Virxe da Xunqueira* respecto al número de camas, de empleados, de consultas primeras y sucesivas, de urgencias y de estancias hospitalarias en ambos periodos de gestión, brutos y agregados por UBAS.

En la Tabla 4 se muestran los resultados de las funciones de producción del hospital *Virxe da Xunqueira* tanto con gestión indirecta como directa. En ambos períodos de gestión, el factor *capital* (número de camas) es significativo al 15% mientras que el factor *producción* (trabajo) no resulta significativo. El análisis de los rendimientos a escala mide la variación de la producción ante un cambio proporcional para los dos factores de producción, y en ambos períodos se observan retornos decrecientes de escala. El signo negativo, tanto de CAM como de TBJ, significa que el producto de la actividad aumenta a un ritmo menor que el incremento de todos los factores. La intersección (tecnología de producción) tiene signo positivo, lo que significa que la tecnología de producción influye positivamente en los incrementos de productividad y es la variable con mayor impacto del modelo en el *output*.

**Tabla 4:** Estimación de las funciones de producción Cobb-Douglas

	Período					
	Fundación pública			Centro gestor SERGAS		
	(gestión indirecta) 2005-2009			(gestión directa) 2011-2015		
	Intersección	CAM	TBJ	Intersección	CAM	TBJ
Coeficiente	85609,61	-5,698	-0,007	105933,7	-9,999	-0,025
t	6,547	-2,380	-0,270	4,854	-2,747	-1,390
p	0,023	0,140	0,812	0,040	0,111	0,299
R^2^		0,765			0,790	
R^2^ ajustada		0,531			0,581	
F		3,264			3,773	
Durbin Watson		1,994			3,087	

CAM: número de camas; TBJ: número de trabajadores del hospital a tiempo completo. El índice de Malmquist para el hospital *Virxe da Xunqueira* indicó que el nivel de eficiencia fue superior tras la reversión al SERGAS (gestión directa) que durante el período como fundación pública (gestión indirecta) (1 vs 0,88082).

## DISCUSIÓN

El método DEA se ha utilizado en varios estudios para evaluar la eficiencia de los hospitales del SERGAS, destacando un estudio que analizó hospitales del SERGAS, aunque ninguno de ellos era fundación pública, que evidencia que los hospitales con mayores dimensiones (número de camas y de trabajadores) muestran rendimientos constantes a escala de la frontera y máxima productividad, mientras que los centros pequeños están en un nivel de rendimientos crecientes a escala, lo que indicaría que sería adecuado aumentar su dimensión^21^. Sin embargo, el hospital *Virxe da Xunqueira* muestra en nuestro estudio retornos decrecientes de escala en los dos escenarios de gestión hospitalaria (como fundación pública y como centro gestor del SERGAS).

Atendiendo al índice de Malmquist, el hospital revertido al SERGAS presenta mayor eficiencia global en su conjunto que cuando era fundación pública. En España destaca un estudio con metodología DEA que compara la eficiencia de hospitales centros gestores del Servicio Andaluz de Salud (SAS) y de hospitales con nuevos modelos de gestión (fundaciones públicas, empresas públicas, concesiones, consorcios)^25^; no compara la eficiencia pre-post reversión al no haberse producido esta durante el periodo de estudio. En dicho periodo, los hospitales con nuevos modelos de gestión fueron más eficientes que los hospitales centro de gestión del SAS (más de un 10% de media), pero se observa una convergencia entre ambos tipos de organizaciones, con un ligero aumento de la eficiencia del 0,50% en el caso de los hospitales centro de gestión del SAS y una disminución de más del 2% en el de los hospitales con nuevos modelos de gestión.

Al contrario que nuestro estudio, otro estudio sustenta la afirmación de que los hospitales con nuevas formas de gestión sanitaria en España son más eficientes que los hospitales centros gestores de diferentes servicios regionales de salud^23^. Tampoco este estudio evalúa el pre-post reversión de las nuevas formas de gestión, y sus conclusiones han sido muy criticadas al no incluir que el número medio de camas en los hospitales centros gestores de los servicios regionales de salud es de 258 y en los de nuevos modelos de gestión de 151, lo que indica que muchos de los hospitales con nuevos modelos de gestión son hospitales comarcales con otro hospital de referencia al que derivar los pacientes. De hecho, el estudio no indica el número de derivaciones de los hospitales con nuevos modelos de gestión a los hospitales de referencia, lo que es fundamental para comprender si se está produciendo una selección de riesgos, que puede impactar significativamente en los resultados en términos de coste, mortalidad, y/o complejidad.

Nuestro estudio muestra que no hay evidencia de mayor eficiencia en los nuevos modelos de gestión (en este caso, fundación pública), a diferencia de un estudio^26^ que, si bien no aborda la comparativa entre la eficiencia pre y post reversión, presenta dos estudios de casos en los que se evalúa la puesta en práctica de los nuevos modelos de gestión sanitaria, la atención primaria en Suecia y los hospitales en España. A falta de cifras comparativas reales, los partidarios de los nuevos sistemas de gestión destacan su mayor eficiencia en el uso de los recursos (camas) y en la maximización del uso de alternativas de atención ambulatoria, con un coste medio por unidad de producción un 30% inferior y una actividad ajustada de recursos humanos un 37% superior; a diferencia de los resultados de nuestro estudio. Sin embargo, esta conclusión no es sorprendente, dado que los nuevos hospitales, de menos de 300 camas, tienden a ser más pequeños en tamaño y personal (y tienen una casuística más sencilla) que los hospitales de gestión tradicional.

Se evaluó mediante la metodología DEA la eficacia de la experiencia de hospitales bajo la figura de concesión y de hospitales centro de gestión del *Servei Valenciá de Salut*, comparando indicadores de rendimiento, coste y calidad para identificar la influencia de la gestión concesionada privada en los resultados^38^. Respecto al rendimiento y la eficiencia, el grupo de hospitales-concesión obtiene buenos resultados, por encima de la media, pero no siempre mejores que los de gestión directa, lo que coincide con los resultados presentados en nuestro trabajo. Ese estudio tampoco comparó los hospitales bajo nuevos modelos de gestión antes y después de su reversión.

Existe un estudio que compara la actividad y la dotación de recursos, antes y después de la reversión a nuevos modelos de gestión hospitalaria (en particular, concesiones administrativas) en la *Comunitat Valenciana*^39^ pero se limita a comparar los datos sin proponer un modelo econométrico que los analice. Dicho estudio indica que el tiempo de espera en el Hospital de La Ribera es de 60 días, dos días menos que en el momento de la reversión hace cinco años y 20 días menos que la media del conjunto de hospitales de la *Comunitat Valenciana*. En el momento de la reversión la espera media en urgencias en el Hospital de La Ribera era de 202 minutos, tres minutos menos que la media. Tras la reversión, se produjo un descenso hasta los 181 minutos.

Las mejoras del modelo de gestión de fundación pública respecto de los parámetros de hospitalización (número de ingresos, estancias, estancia media y porcentaje de ocupación) podrían deberse al modelo de financiación de la fundación pública mediante un sistema de pago basado en Grupo Relacionados por el Diagnóstico (GRD) para cada paciente atendido, independientemente de cuánto dinero gastó realmente el hospital en su tratamiento. Los mejores resultados en cuanto a consultas y urgencias pueden deberse a una menor derivación de consultas de la Atención Primaria al hospital fundación pública y una mayor derivación de pacientes de urgencias del hospital fundación pública al hospital de referencia del SERGAS. No obstante, estas hipótesis no han sido evaluadas en nuestro trabajo por no disponer de dichos datos. Por otro lado, la gestión directa resulta notable en lo que respecta a intervenciones quirúrgicas y lista de espera, incluyendo el número de pacientes y el tiempo de espera promedio. Es interesante destacar que las diferencias son mínimas en algunos casos, como puede ser en cuanto a consultas, urgencias o ingresos, lo que sugiere, en general, resultados positivos en ambos modelos de gestión. Un meta-análisis sobre hospitales chinos destacó el efecto positivo del pago por GRD en el tiempo de espera medio, pero no así en las tasas de mortalidad o readmisión de los pacientes ^40^. Otro estudio realizado en Hong Kong con una muestra notablemente amplia de pacientes analizó la relación entre el sistema GRD y diferentes variables de interés (como tiempo de espera medio o volumen de pacientes ingresados). Las listas de espera se redujeron al mismo tiempo que incrementó la congestión en los hospitales públicos; también se observó cierta reducción de la mortalidad hospitalaria, pero no hay suficiente evidencia para relacionarla con la aplicación del pago por GRD ^41^. Un estudio israelí también observó disminución de la estancia media hospitalaria asociada a un sistema de pago por GRD; los autores resaltaron la dificultad de comparación y aplicación de este tipo de estudios en otros países y contextos ^42^. Como se puede observar, los resultados de estos tres estudios se asemejan a los obtenidos en el nuestro, resaltando las diferencias mínimas entre ambas formas de gestión y las ligeras mejoras técnicas y de eficiencia al aplicarse el pago por GRD.

Se ha observado una menor producción de consultas externas y urgencias en el hospital *Virxe da Xunqueira* como centro gestor del SERGAS (gestión directa) que como hospital fundación (indirecta), y ello puede ser debido a que una parte de esa atención sería realizada en mayor medida por la Atención Primaria. Este resultado discrepa con otro estudio que encontró una tasa de relevo ambulatorio superior (una mayor actividad de consultas de Atención Primaria) en la gestión por concesión que en los centros de gestión directa ^43^.

Salvador Peiró sugirió en 2013 que el menor coste por ingreso en las concesiones respecto de los hospitales de gestión directa se relaciona con un mayor número de ingresos al repartirse los costes fijos ^44^. Eso mismo parecen sugerir los resultados de nuestro DEA, que muestran una mejora en la eficiencia para las variables de ingresos y estancia media para el modelo de gestión indirecta (fundación) comparado con el modelo de gestión directa. También otro estudio realizado con 1.604 hospitales encontró que la estancia media era significativamente menor en las concesiones que en los hospitales de gestión directa ^45^.

El hospital *Virge da Xunqueira* presentó una mayor eficiencia en el área quirúrgica con gestión directa, lo que redunda en mayor eficiencia en los indicadores número de pacientes en lista de espera quirúrgica y tiempo medio de espera para una intervención de cirugía. Una tesis doctoral de 2014 constató, mediante un análisis de regresión de los pacientes equivalentes frente al coste, que las concesiones fueron más eficientes en el área quirúrgica que la gestión directa, excepto un hospital concesionario que sería eficiente en el área médica respecto a la media del conjunto de hospitales ^46^.

El presente trabajo presenta limitaciones propias de la metodología DEA: la exclusión de variables no consideradas puede llevar a identificar ineficiencias espurias; el DEA es muy adecuado para evaluar la eficiencia (o ineficiencia) relativa pero no la absoluta cuando el objetivo es alcanzar resultados potenciales o ideales; y al tratarse de un método no paramétrico, es difícil formular pruebas estadísticas de hipótesis. También cabe destacar la opacidad y falta de transparencia del organismo regulador pertinente, una limitación evidente para la obtención de los datos de interés en la post-integración.

El hospital *Virge da Xunqueira* presentó mayor eficiencia en las áreas de consultas, atención a urgencias, pacientes hospitalizados e indicadores relacionados con la estancia como fundación pública (gestión indirecta), mientras que fue más eficiente en intervenciones quirúrgicas y lista de espera como centro gestor del SERGAS (gestión directa). Además, los factores de producción presentaron retornos decrecientes a escala en ambos tipos de gestión. Por lo tanto, en este estudio no se obtuvo evidencia suficiente para afirmar que los nuevos modelos de gestión sean más eficientes que la gestión directa.

## Data Availability

Se encuentran disponibles bajo petición al autor de correspondencia.

## ANEXO 1

Datos empleados en la estimación de la función de producción, brutos y ponderados por unidades básicas de atención sanitaria

**Table:**

	Fundación pública				
	2005	2006	2007	2008	2009
*Datos brutos*					
*Inputs*					
Número de camas	76	76	74	74	75
Número de empleados	274	301	264	284	326
*Outputs*					
Consultas primarias	26.211	25.631	25.670	24.837	22.485
Consultas sucesivas	45.208	37.344	42.132	41.273	39.009
Urgencias	16.380	17.498	18.054	17.872	17.458
Estancias	20.154	20.504	21.254	22.354	23.385
*Datos ponderados por Unidad Básica Asistencial (UBAS)*					
*Inputs*					
Número de camas	5.776	5.776	5.476	5.476	5.625
Número de empleados	75.076	90.601	69.696	80.656	106.276
*Outputs*					
Consultas primarias	13.105	12.815	12.835	12.419	11.243
Consultas sucesivas	11.302	9.336	10.533	10.318	9.752
Urgencias	8.190	8.749	9.027	8.936	8.729
Estancias	20.154	20.504	21.254	22.354	23.385
Centro gestor del Servicio Gallego de Salud					
	2011	2012	2013	2014	2015
*Datos brutos*					
*Inputs*					
Número de camas	75	76	76	76	74
Número de empleados	328	304	304	376	375
*Outputs*					
Consultas primarias	23.355	23.321	22.195	21.391	21.433
Consultas sucesivas	39.222	39.614	40.257	40.651	42.145
Urgencias	16.413	15.386	15.671	15.819	16.727
Estancias	17.845	16.020	17.454	15.801	17.749
*Datos ponderados por Unidad Básica Asistencial (UBAS)*					
*Inputs*					
Número de camas	5.625	5.776	5.776	5.776	5.476
Número de empleados	107.584	82.944	92.416	141.376	140.625
*Outputs*					
Consultas primarias	11.678	11.661	11.098	10.696	10.717
Consultas sucesivas	9.806	9.904	10.064	10.163	10.536
Urgencias	8.207	7.693	7.836	7.910	8.364
Estancias	17.845	16.020	17.454	15.801	17.749
